# Thrombotic Thrombocytopenic Purpura Without Neurological Involvement: A Case Report and Review of the Diagnostic and Treatment Strategies

**DOI:** 10.7759/cureus.83654

**Published:** 2025-05-07

**Authors:** Kapeel Daive, Jegadis Sreeneyasan

**Affiliations:** 1 Medicine, Lancashire Teaching Hospitals NHS Foundation Trust, Preston, GBR; 2 Medicine, Royal Bolton Hospital, Bolton, GBR

**Keywords:** adamts13, caplacizumab, microangiopathic hemolytic anemia, plasma exchange therapy, plasmic score, thrombotic thrombocytopenic purpura, von willebrand factor

## Abstract

This case report describes the presentation of thrombotic thrombocytopenic purpura (TTP) without neurological involvement in a 48-year-old woman. TTP is a relatively rare, life-threatening condition that comprises thrombotic microangiopathy (TMA) and enzymatic dysfunction of ADAMTS13 (a disintegrin-like metalloproteinase with thrombospondin motif type 1, member 13, which regulates platelet aggregation). Another similar condition that presents with TMA in adults is atypical hemolytic uremic syndrome. It is challenging to differentiate between the two conditions clinically, especially in the absence of neurological symptoms. However, plasma exchange is the initial lifesaving treatment for both in acute situations. Our patient had a history of bruising, which was evident in clinical examination, without any other positive findings. However, ADAMTS13 activity was 0%, and the platelet count was 4 × 10⁹/L on arrival. Timely investigation and treatment not only saved her life but also reduced end-organ damage. This report provides an overview of the acute presentation of TTP, positive clinical findings, investigations, diagnosis, treatment, and post-treatment outcomes.

## Introduction

Thrombotic thrombocytopenic purpura (TTP) is a sudden onset of hemolytic microangiopathy, usually presenting with a pentad of symptoms, namely, fever, hemolysis, thrombocytopenia, impaired renal function, and neurological abnormalities [1]. In adults, TTP is almost always acquired and immune-mediated [1]. Antibodies are produced against von Willebrand factor (VWF)-cleaving protease (ADAMTS13), resulting in reduced cleavage of large, active VWF molecules into smaller, inactive units. This leads to platelet activation, microthrombi formation, and dysfunction of vital organs, typically affecting the kidneys and nervous system. Although the exact trigger for TTP is not well understood, pregnancy, antiplatelet drugs, birth control pills, immunosuppressive agents, and HIV are commonly associated [2]. Atypical hemolytic uremic syndrome (aHUS) is usually caused by uncontrolled complement activation in the alternative pathway, resulting in thrombotic microangiopathy (TMA) with hemolysis and end-organ dysfunction, primarily affecting the kidneys.

## Case presentation

A 48-year-old female was referred by her general physician to the Same Day Emergency Care unit. She presented with a four-day history of feeling generally unwell, lethargy, bruising around her left breast and elbow, and dark urine. There was no history of trauma, fever, abdominal pain, diarrhea, or lower urinary tract symptoms. The patient had a past medical history of mild psoriasis, hypermobility, and chronic lower back pain, for which she regularly took amitriptyline. She denied any history of allergies. She worked in an office and lived alone. She was an active smoker with a smoking history of 20-pack-years and consumed alcohol socially. Her physical examination was unremarkable, except for bruising around the pressure areas of her joints. Urgent blood tests, including a coagulation profile, were requested (Table 1).

**Table 1:** Investigation results. Platelet count is severely low with moderately high inflammatory markers. Blood film decisively reflects hemolysis with thrombocytopenia; however, there is no drop in hemoglobin or rise in reticulocytosis. It is noticeable that ADAMTS-13 activity is 0%, which is sufficient to diagnose TTP. Moderate renal function impairment is not uncommon in TTP. Normal prothrombin time and partial thromboplastin time rule out disseminated intravascular coagulation. ESR: erythrocyte sedimentation rate; ADAMTS 13: a disintegrin and metalloproteinase with thrombospondin type 1 motif, 13; TTP: thrombotic thrombocytopenic purpura

Investigation	Result	Normal value
Hemoglobin	118	115–165 g/L
White blood cell	7.4	4–11 × 10^9^/L
Platelet	4	150–450 × 10^9^/L
Mean cell volume	83	
Urea	20	2.5–7.8 mmol/L
Creatinine	189	45–84 mmol/L
C-reactive protein	85	0–5 mg/L
ESR	45	0–15 mm/hour
Reticulocyte	77.6	22–80 × 10^9^/L
Bilirubin	31	0–21 µmol/L
Prothrombin time	10.1	9.9–11.1 seconds
International normalized ratio	1.0	
Partial thromboplastin time	21.4	21–30 seconds
Complement C3	2.5	0.75–1.65 g/L
Complement C4	0.42	0.14–0.54 g/L
Urine protein creatinine ratio	80	0–30 mg/mmol
Blood film	Red blood cells showed anisocytosis with tear and drop cells. Platelets are reduced in number without clumping	
Direct Coombs test	Negative	
ADAMTS-13	0% (normal value: 50–160%)	

We discussed the case with a hematologist for an urgent blood film review, which confirmed hemolysis with thrombocytopenia, consistent with TTP or aHUS. The PLASMIC score was 7, reflecting a 72% risk of severe ADAMTS13 deficiency. An urgent ADAMTS13 test was sent, which later showed 0% enzymatic activity. She was admitted to the intensive care unit for urgent plasma exchange. Intravenous methylprednisolone, rituximab, and caplacizumab were administered as part of her treatment.

Her ADAMTS13 activity improved to 39%, and her platelet count increased to 150 × 10⁹/L. She was discharged with outpatient hematology follow-up. Blood test results during follow-up showed significant improvement in renal function, with an estimated glomerular filtration rate of 68, up from 28 at admission, and ADAMTS13 activity improved to 74%, with normal platelet and hemoglobin levels.

## Discussion

TTP is a critical condition with a mortality rate of >90% if left untreated [2]. This rare disease has an incidence of 1 to 13 cases per million individuals, varying by geographic region [2]. Early recognition and initiation of treatment are key factors in reducing mortality [2]. It is a type of microangiopathic hemolytic anemia caused by dysfunction of VWF due to the formation of antibodies against the cleaving enzyme ADAMTS13. ADAMTS13 is a protease that acts on multimers of VWF, breaking them into smaller monomers. Deficiency in ADAMTS13 activity results in ultra-large active VWF units, which lead to platelet aggregation. This platelet aggregation causes thrombocytopenia and the formation of microthrombi, which, along with fibrin, occlude small vessels, leading to the fragmentation of red cells and platelets. Microthrombi in small vessels also cause ischemia in vital organs such as the kidneys.

TTP typically presents with a pentad of symptoms, namely, fever, hemolysis, thrombocytopenia, impaired renal function, and neurological manifestations, such as headache, confusion, seizures, or stroke [2]. However, some patients may not exhibit all symptoms. In our case, the patient had no fever or neurological manifestations, which could be mistaken for aHUS. aHUS is generally caused by unregulated complement activation and presents with a triad of microangiopathic hemolysis, thrombocytopenia, and abnormal renal function.

Laboratory assessment is essential in diagnosing TTP due to the variability of symptoms and the risk of delayed organ damage. To confirm microangiopathic hemolysis and thrombocytopenia, full blood counts, a peripheral blood film, reticulocyte count, bilirubin, haptoglobin, and lactate dehydrogenase levels are required. MRI of the brain, renal function tests, and liver function tests help assess organ dysfunction. A normal coagulation profile helps rule out disseminated intravascular coagulation, another cause of TMA [3]. The gold standard for diagnosis is measuring ADAMTS13 activity, with levels below 5% confirming TTP; however, test results can take two to three days, delaying diagnosis and reducing survival chances [4]. In such cases, the PLASMIC score is recommended to predict the likelihood of ADAMTS13 activity being ≤10% [5]. In our case, the PLASMIC score was 7 (international normalized ratio: <1.5, platelet: <30, mean corpuscular volume: <90, creatinine: <178, hemolysis positive), reflecting a 72% probability of severe ADAMTS13 deficiency [5].

Plasma exchange combined with high-dose corticosteroids is the initial and most critical treatment for TTP [5]. Plasma exchange effectively removes ultra-large VWF multimers and antibodies against ADAMTS13 from circulation [5]. Corticosteroids suppress the reticuloendothelial system and reduce antibody formation. Anti-CD20 therapy (rituximab) is highly effective in suppressing antibody production [3]. Caplacizumab, another monoclonal antibody, is a potent treatment that targets the A1 domain of VWF, preventing platelet adhesion. It rapidly halts microthrombi formation, though bleeding is a serious potential side effect [6].

## Conclusions

TTP is a medical and hematological emergency with a high risk of poor outcomes if the diagnosis is not considered or worked up appropriately. A peripheral blood film is a key investigation in cases of acute thrombocytopenia to rule out microangiopathic hemolysis. Similarly, the use of the PLASMIC score can increase confidence in initiating treatment for TTP, even when there is a delay in receiving ADAMTS13 results. Early involvement of relevant specialists, including hematologists, neurologists, nephrologists, and intensivists, is crucial for providing optimal care and improving patient outcomes.
